# Supplementary material for: Climate and urbanization drive changes in the habitat suitability of Schistosoma mansoni competent snails in Brazil
Source: Nat Commun. 2024 Jun 19;15:4838. doi: 10.1038/s41467-024-48335-9 (PMC11186836; doi:10.1038/s41467-024-48335-9)
Supplement: Supplementary file 3 — Reporting Summary [file 41467_2024_48335_MOESM3_ESM.pdf]

nature portfolio

Corresponding author(s): Caroline Glidden

Last updated by author(s): March 14, 2024

Reporting Summary

Nature Portfolio wishes to improve the reproducibility of the work that we publish. This form provides structure for consistency and transparency in reporting. For further information on Nature Portfolio policies, see our Editorial Policies and the Editorial Policy Checklist.

Statistics

For all statistical analyses, confirm that the following items are present in the figure legend, table legend, main text, or Methods section.

n/a	Confirmed	
☐	☒	The exact sample size (*n*) for each experimental group/condition, given as a discrete number and unit of measurement
☐	☒	A statement on whether measurements were taken from distinct samples or whether the same sample was measured repeatedly
☒	☐	The statistical test(s) used AND whether they are one- or two-sided<br>*Only common tests should be described solely by name; describe more complex techniques in the Methods section.*
☐	☒	A description of all covariates tested
☒	☐	A description of any assumptions or corrections, such as tests of normality and adjustment for multiple comparisons
☐	☒	A full description of the statistical parameters including central tendency (e.g. means) or other basic estimates (e.g. regression coefficient) AND variation (e.g. standard deviation) or associated estimates of uncertainty (e.g. confidence intervals)
☒	☐	For null hypothesis testing, the test statistic (e.g. *F*, *t*, *r*) with confidence intervals, effect sizes, degrees of freedom and *P* value noted<br>*Give P values as exact values whenever suitable.*
☒	☐	For Bayesian analysis, information on the choice of priors and Markov chain Monte Carlo settings
☒	☐	For hierarchical and complex designs, identification of the appropriate level for tests and full reporting of outcomes
☒	☐	Estimates of effect sizes (e.g. Cohen's *d*, Pearson's *r*), indicating how they were calculated

Our web collection on statistics for biologists contains articles on many of the points above.

Software and code

Policy information about availability of computer code

Data collection	Biomphalaria occurrence data was collected by the São Paulo Health Secretariat and Fio Cruz-Minas Gerais. Background occurrence points were downloaded from the Global Biodiversity Information Facility using the R package rgbif. All freshwater, terrestrial animals were included in the download, with species names retrieved from the IUCN.
Data analysis	R version 4.3.2, Python via Google Co-Lab, and Google Earth Engine were used to run the analyses. All code is available on https://github.com/ckglidden/biomphalaria-sdm-brazil .<br><br>We used the following R packages that are also cited in the manuscript: Xgboost, blockCV, bayesianOptimization, pROC, caret, SHAPforxgboost, pdp, ggplot2, raster, and geobr. We used the Google Earth Engine API in Python.

For manuscripts utilizing custom algorithms or software that are central to the research but not yet described in published literature, software must be made available to editors and reviewers. We strongly encourage code deposition in a community repository (e.g. GitHub). See the Nature Portfolio guidelines for submitting code & software for further information.

Data

Policy information about availability of data

All manuscripts must include a data availability statement. This statement should provide the following information, where applicable:

- Accession codes, unique identifiers, or web links for publicly available datasets
- A description of any restrictions on data availability
- For clinical datasets or third party data, please ensure that the statement adheres to our policy

Data is available on https://github.com/ckglidden/biomphalaria-sdm-brazil/ and via Zenodo using the doi 10.5281/zenodo.10975612.

We obtained data from CHELSA (https://chelsa-climate.org/downloads/), WorldPop (https://developers.google.com/earth-engine/datasets/catalog/WorldPop_GP_100m_pop), Global Human Settlement Layer (https://human-settlement.emergency.copernicus.eu/ghs_pop2019.php), MAPBIOMAS (https://brasil.mapbiomas.org/en/), JRC Global Surface Water Mapping Layers (https://developers.google.com/earth-engine/datasets/catalog/JRC_GSW1_4_GlobalSurfaceWater), Merit Hydro: Global Hydrography Dataset (https://developers.google.com/earth-engine/datasets/catalog/MERIT_Hydro_v1_0_1), NASADEM: NASA Digital Elevation (https://developers.google.com/earth-engine/datasets/catalog/NASA_NASADEM_HGT_001), WWF HydroSheds Free Flowing River Networks v1(https://developers.google.com/earth-engine/datasets/catalog/WWF_HydroSHEDS_v1_FreeFlowingRivers), OpenLandMap Soil Properties (clay: https://developers.google.com/earth-engine/datasets/catalog/OpenLandMap_SOL_SOL_CLAY-WFRACTION_USDA-3A1A1A_M_v02; sand: https://developers.google.com/earth-engine/datasets/catalog/OpenLandMap_SOL_SOL_SAND-WFRACTION_USDA-3A1A1A_M_v02; water: https://developers.google.com/earth-engine/datasets/catalog/OpenLandMap_SOL_SOL_WATERCONTENT-33KPA_USDA-4B1C_M_v01; carbon: https://developers.google.com/earth-engine/datasets/catalog/OpenLandMap_SOL_SOL_ORGANIC-CARBON_USDA-6A1C_M_v02; pH: https://developers.google.com/earth-engine/datasets/catalog/OpenLandMap_SOL_SOL_PH-H2O_USDA-4C1A2A_M_v02; bulk density: https://developers.google.com/earth-engine/datasets/catalog/OpenLandMap_SOL_SOL_BULKDENS-FINEEARTH_USDA-4A1H_M_v02).

Research involving human participants, their data, or biological material

Policy information about studies with human participants or human data. See also policy information about sex, gender (identity/presentation), and sexual orientation and race, ethnicity and racism.

Reporting on sex and gender	N/A
Reporting on race, ethnicity, or other socially relevant groupings	N/A
Population characteristics	N/A
Recruitment	N/A
Ethics oversight	N/A

Note that full information on the approval of the study protocol must also be provided in the manuscript.

Field-specific reporting

Please select the one below that is the best fit for your research. If you are not sure, read the appropriate sections before making your selection.

☒ Life sciences ☐ Behavioural & social sciences ☐ Ecological, evolutionary & environmental sciences

For a reference copy of the document with all sections, see nature.com/documents/nr-reporting-summary-flat.pdf

Life sciences study design

All studies must disclose on these points even when the disclosure is negative.

Sample size	First, our original dataset contained 11k snail occurrence records and 55K occurrence records for background points. We reduced the snail dataset by selecting only the focal Biomphalaria species, and then thinned the dataset so that we only selected one point per location so to eliminate issues with pseudo-replication and auto-correlation. The background set was reduced by selecting 2x the number of occurrence points from a background mask. The background mask was created so that the probability of selection was proportional to the number of records recorded in the same location.
Data exclusions	We excluded Biomphalaria data points that occurred in the same location ("thinned" the dataset) and reduced the number of background points to 2x the number of occurrence points so adequatley account for the sampling landscape without creating an unbalanced dataset.
Replication	We provide the entire code pipeline on our github so that the entire study can be reproduced. We also included bootstrapping in each step of our machine learning analysis so to quantify uncertainty around our results.
Randomization	There were no group assignments as a part of the study design so randomization was not necessary.

Blinding	Blinding was not relevant to our study as knowing information related to the study outcome did not have the potential to influence study results.

Reporting for specific materials, systems and methods

We require information from authors about some types of materials, experimental systems and methods used in many studies. Here, indicate whether each material, system or method listed is relevant to your study. If you are not sure if a list item applies to your research, read the appropriate section before selecting a response.

Materials & experimental systems

n/a	Involved in the study	
☒	☐	Antibodies
☒	☐	Eukaryotic cell lines
☒	☐	Palaeontology and archaeology
☐	☒	Animals and other organisms
☒	☐	Clinical data
☒	☐	Dual use research of concern
☒	☐	Plants

Methods

n/a	Involved in the study	
☒	☐	ChIP-seq
☒	☐	Flow cytometry
☒	☐	MRI-based neuroimaging

Animals and other research organisms

Policy information about studies involving animals; ARRIVE guidelines recommended for reporting animal research, and Sex and Gender in Research

Laboratory animals	N/A
Wild animals	N/A
Reporting on sex	N/A
Field-collected samples	Occurrence points and morphology of invertebrate Biomphalaria were collected in the field, and some tissue was isolated for genetic analyses for species identification. This did not require maintaining the animals in the laboratory environment.
Ethics oversight	N/A

Note that full information on the approval of the study protocol must also be provided in the manuscript.

Plants

Seed stocks	*Report on the source of all seed stocks or other plant material used. If applicable, state the seed stock centre and catalogue number. If plant specimens were collected from the field, describe the collection location, date and sampling procedures.*
Novel plant genotypes	*Describe the methods by which all novel plant genotypes were produced. This includes those generated by transgenic approaches, gene editing, chemical/radiation-based mutagenesis and hybridization. For transgenic lines, describe the transformation method, the number of independent lines analyzed and the generation upon which experiments were performed. For gene-edited lines, describe the editor used, the endogenous sequence targeted for editing, the targeting guide RNA sequence (if applicable) and how the editor was applied.*
Authentication	*Describe any authentication procedures for each seed stock used or novel genotype generated. Describe any experiments used to assess the effect of a mutation and, where applicable, how potential secondary effects (e.g. second site T-DNA insertions, mosiacism, off-target gene editing) were examined.*
